# Pneumococcal Serotypes Colonise the Nasopharynx in Children at Different Densities

**DOI:** 10.1371/journal.pone.0163435

**Published:** 2016-09-29

**Authors:** Fernanda Rodrigues, Leon Danon, Begonia Morales-Aza, Paulina Sikora, Valtyr Thors, Muriel Ferreira, Katherine Gould, Jason Hinds, Adam Finn

**Affiliations:** 1 Hospital Pediátrico Coimbra, Centro Hospitalar e Universitário de Coimbra, Coimbra, Portugal; 2 Faculty of Medicine, Universidade de Coimbra, Coimbra, Portugal; 3 School of Social and Community Medicine, University of Bristol, Bristol, United Kingdom; 4 School of Cellular and Molecular Medicine, University of Bristol, Bristol, United Kingdom; 5 Institute for Infection and Immunity, St. George's, University of London, London, United Kingdom; 6 BUGS Bioscience, London Bioscience Innovation Centre, London, United Kingdom; Universidade de Lisboa Faculdade de Medicina, PORTUGAL

## Abstract

Prevalence of pneumococcal serotypes in carriage and disease has been described but absolute serotype colonisation densities have not been reported. 515 paediatric nasal swab DNA extracts were subjected to lytA qPCR and molecular serotyping by microarray. Absolute serotype densities were derived from total pneumococcal density (qPCR cycle threshold and standard curve) and relative abundance (microarray) and varied widely. Compared to all serotype densities observed, the strongest evidence of differences was seen for serotypes 21 and 35B (higher) and 3, 38 and non-typeables (lower) (p<0.05) with a similar hierarchy when only a single serotype carriage was assessed. There was no evidence of any overall density differences between children with single or multiple serotypes detected but serotypes with mid-range densities were more prevalent. The hierarchy of distinct pneumococcal serotype carriage densities described here for the first time, may help explain the dynamics of transmission between children.

## Introduction

Much attention has been devoted to differences in prevalence in carriage of pneumococcus (Sp) and carriage of specific serotypes and how these correlate with prevalence in disease [1].

In the pre-pneumococcal conjugate vaccine (PCV) era, the rank order of serotypes found in colonisation was reasonably stable across different populations with certain disease-causing serotypes like serotypes 1 and 5 rarely detected [1,2]. Subsequent studies have suggested that the diversity and overall relative prevalences of pneumococcal serotypes revert to a particular population structure following the perturbation caused by universal childhood PCV implementation [3,4]. Weinberger et al. [5] showed an association between increased carriage prevalence and resistance to non-opsonic neutrophil-mediated killing: the more prevalent serotypes, such as 19F and 23F being most resistant, while types rarely isolated, such as 4 and 5, were more efficiently killed. Resistant serotypes tended to be more heavily encapsulated. An association between polysaccharide structure and carriage prevalence was also identified with more common serotypes having less energy expended on capsule generation [5]. Other microbial factors, such as adhesins, toxins and proteins that avoid host immune effectors, may also influence carriage prevalence of specific strains [6].

The tendency of certain serotypes to be undetectable in the nasopharynx may also be associated with their low density and/or duration of carriage. Wide ranges of bacterial DNA concentrations in nasopharyngeal samples from healthy pre-school children carrying pneumococcus have been shown using quantitative (q)PCR [7]. That simultaneous carriage of multiple Sp serotypes occurs has long been known [8], but recent technical advances, notably microarray detection, has made it feasible both to detect them and evaluate their relative abundance in a sample [9]. Wyllie and colleagues published evidence suggesting that children colonised with more Sp serotypes have higher overall carriage density than those with fewer [10]. Taken together, these findings suggest that the well known serotype hierarchy of prevalence in carriage at the population level may overlie another hierarchy within individual children, with certain serotypes tending to be more abundant (and thus easily detected) than others.

## Material and Methods

In March 2011 we took nasopharyngeal swabs from 515 healthy pre-school children in daycare in Coimbra, Portugal. The study was approved by the Ethics Committee of Centro Hospitalar de Coimbra and parents provided written informed consent for their child to participate. The median age was 40 months (range 5–84 months) and 54% were male. Swabs were placed in 2mL cryovials containing 1.5mL soya tryptone glucose glycerine (STGG) transport and storage medium. Although the samples are taken into STGG, raising the theoretical possibility of proliferation after sampling, in practice these samples were placed into a cold box at 4°C immediately after collection and then frozen at -80°C within a few hours, so that very little if any bacterial division is likely to have occurred. After storage at -80°C, DNA extracts were prepared from 340μL medium and from colistin blood agar lawn cultures of 50μL medium and subjected respectively to *lytA* qPCR as previously described [7]. Molecular serotyping was performed by microarray (Senti-SP v1.4, BUGS Bioscience, London, UK; http://bugsbio.org) in order to detect and quantify DNA from each serotype present [11]. Culturing STGG prior to DNA extraction and microarray increases yields of pneumococcal DNA and thus sensitivity of detection of serotypes present at low abundance [12]. The microarray determines serotype(s) present in the sample based on the combination of capsule polysaccharide biosynthesis (*cps*) genes detected and assigns relative abundance using the signal intensity of *cps* genes associated with each serotype [13]. Additionally, detection of non-encapsulated non-typeable (NT) pneumococcal lineages lacking *cps* genes and discrimination of closely related streptococcus species, such *S*. *mitis*, *S*. *oralis and S*. *pseudopneumoniae* that may contain *cps* gene homologues, is possible.

A standard curve created using a log-phase reference strain broth culture was used to convert *lytA* qPCR cycle threshold (*Ct*) values into total Sp density in gene copies/mL as previously reported [7]. Absolute densities were calculated as follows: for those samples where a single serotype is present, the absolute density is simply the total density of Sp measured by PCR; where more than one serotype were isolated, the total density was adjusted according to the relative abundance of each serotype as obtained by microarray. The distribution of absolute density for all serotypes was fitted to a log-normal distribution using maximum likelihood estimation implemented in the R package ‘fitdistrplus’ (S1 Fig). Each individual serotype distribution was log-transformed and compared to the log-transform of all serotypes using a two-sided Welch’s t-test. The results were confirmed with a Kolmogorov-Smirnov non-parametric test on the absolute values (not log-transformed). All analyses were performed in R version 3.2.3 (2015-12-10), using the following packages: ggplot2 (2.0.0), fitdistrplus (1.0–6) [14].

## Results

Sp was detected by *lytA* qPCR in 337/515 swabs (65.4%), 299 of which showed Sp and/or closely related species bearing *cps* gene homologues detected by microarray.

**Fig 1:**
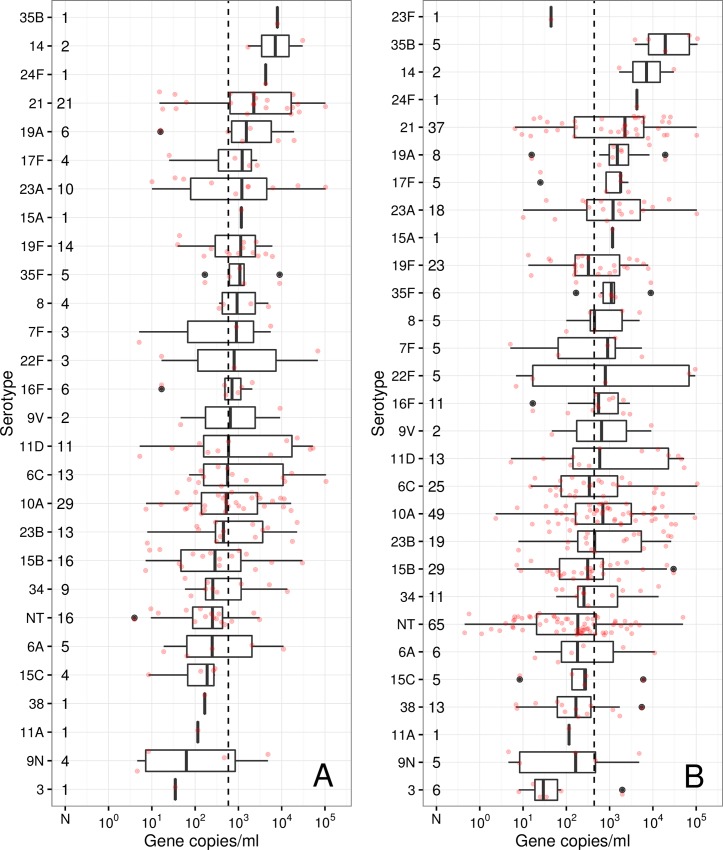
Serotype against observed absolute densities of pneumococcal serotypes. Serotype against observed absolute density for samples where a single serotype or strain was identified in the microarray (A) and for all samples (B). The red dots denote individual measurements of density. In the box and whisker plots, for each serotype, the central line denotes the median of the distribution, the box edges denote upper and lower quartiles and the whiskers extend 1.5 times the interquartile range past the quartiles. The black dots denote outliers (also shown in red), and the dotted vertical line marks the overall median as a guide to the eye. Numbers of measurements for each serotype are shown to the right of the y axis. Boxplots are ordered by median value, from lowest (bottom) to highest (top) of the solo serotypes in both panels.

The absolute density of Sp, *X*, has a range over 5 orders of magnitude, between 0.45–107148, and is well described by a log-normal distribution, *X*∼ln*N*(*μ*,*σ*^2^), with *μ* = 6.10 (95% CI 5.98–6.22) and *σ* = 2.43 (95% CI 2.35–2.52), giving a median of absolute density of 447.9 gene copies/mL (95% CI 395.5–507.2). 31 distinct serotypes plus non-typeables (NT) were identified among 400 isolates. The number of children (out of 299) carrying only one Sp serotype (or NT) was 210, carrying two was 77, 3 was 8 and 4 was 4. The absolute density, grouped by serotype, for the 210 single serotype carriers is shown in the Fig 1A, with the median density of the whole ensemble plotted as a guide to the eye (vertical dotted line). To test the hypothesis that the apparent differences between serotype densities were unlikely to have occurred by chance, we compared each serotype with all the observations including NTs combined together. The strongest evidence of differences was seen for serotypes 21 and and non-typeables (lower) (p<0.05 t-test and KS test). All serotypes, including instances of multiple serotype carriage are shown in the same order in Fig 1B. In this larger dataset there is also strong evidence of differences for 35B (higher) and 3 and 38 (lower) (p<0.05 t-test and KS test). For several others, although there were too few instances to power a comparison at p<0.05, the apparent differences were often large, with 10A, 35F and 23A showing higher density with p<0.1.

To confirm the results of the statistical tests, we also performed the following permutation-based test. The vector of serotype labels was shuffled and the mean serotype density for each (shuffled) serotype was computed. This procedure was repeated 10,000 times and the actual (real sample) mean for was compared to the distribution of 10,000 shuffled mean values, computing its quintile. We found that the mean density of serotypes 21 and 35B fell above the 95^th^ percentile of the shuffled mean distribution, and the mean density for serotype 3 and 38 fell below the 5^th^ percentile of the shuffled mean distribution. This further supports the results of the KS test and t-tests.

**Fig 2:**
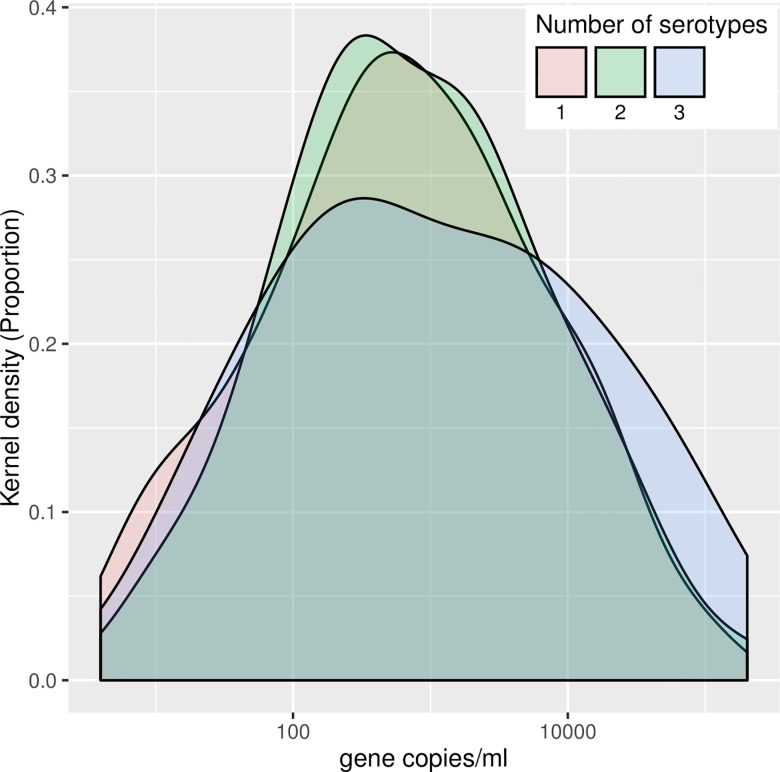
Distribution of carriage density for serotype. Kernel Density plot showing the distribution of density plotted for different numbers of distinct serotypes recovered per child. The density of carriage does not differ with the number of serotypes found.

In contrast to the only previous study to examine this [10], no evidence was found for any relationship between total absolute density of Sp and the number of serotypes detected in each sample (Fig 2).

**Fig 3:**
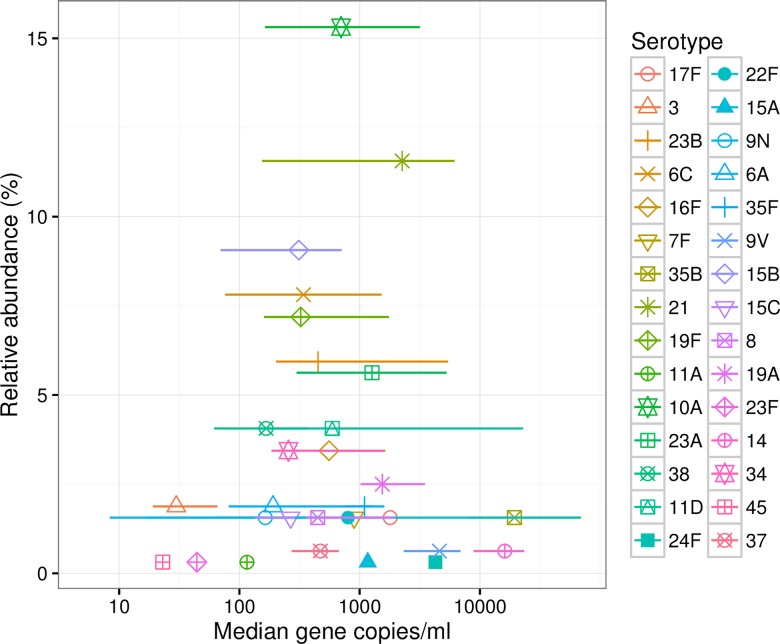
Serotype density against relative abundance. Serotype density against observed relative abundance. For each serotype, the median density is plotted against the relative abundance as a percentage of the entire sample, and horizontal lines denote interquartile ranges. Coloured symbols denote individual serotypes.

To clarify the relationship between serotype density and colonisation potential, serotype frequency (the relative abundance of a particular serotype in the population) is shown as a function of median serotype density, for all samples (Fig 3).

Those serotypes showing an intermediate density range (median between 400–8,000 gene copies/mL) include those that are most abundant in the population unlike serotypes that appear at low densities, or more surprisingly, serotypes colonising at high density. Thus our data suggest that intermediate density may be a necessary condition for high prevalence in the population, but not a sufficient one.

## Discussion

The observation that certain disease causing serotypes are very rarely detected in carriage has led to speculation that they are usually present with others and only at low densities. Our data suggest, for the first time, that a hierarchy of serotype carriage densities may exist. Although the culture enrichment step used may have amplified different strains present together to a different extent, microarray results from culture-amplified mixtures of serotypes have been shown to correlate well with uncultured mixtures [9] and differences were also evident amongst samples with only a single serotype detected. Given the sampling error and technical limitations that must inevitably commonly lead to failure to detect organisms that are present in low abundance and not swabbed or which fall below detection limits, these results suggest that pneumococci and closely genetically related bacteria usually colonise young children and are perhaps transmitted between them as a complex mixed community rather than as a single strain.

The prevalences of all pneumococcal serotypes in carriage decline uniformly with increasing age in pre-school children [15] and subsequent modelling proposes an erosion of serotype-specific selective advantage by putative serotype-non-specific anti-protein immune responses [16]. Our results show a slight downward trend in overall pneumococcal density with increasing age, which is not statistically significant (data not shown). However, it remains theoretically possible that the apparent differences in carriage density of different serotypes we show here could be due, at least in part, to differential age-related effects on different serotypes.

If a quantitative hierarchy of density between serotypes normally exists, it is possible that imbalances induced, for example, by acquisition of new strains or species of bacteria, immune responses to vaccines and other changes in host immunity, intercurrent viral infections, antibiotic use or exposure to environmental smoke, could predispose to mucosal symptomatic or invasive infection. In addition, it is likely that a complex interplay of bacterial and host factors affects the population structure [16].

Accordingly, studies of colonisation in disease and in the presence of these other risk factors, examining serotypes and strain density are warranted.

These observations also call into question the validity of continuing to focus on colonising bacteria as individual species, defined by highly selected and somewhat arbitrary phenotypic features relevant to *in vitro* culture more than *in vivo* biology. Such categorisation ignores the natural tendency of bacteria to co-exist and evolve across these boundaries. This is exemplified by the detection of numerous closely related Streptococcus species in this dataset, which bear homologues of the Sp *cps* genes [17]. One can debate whether or not to include them in analyses when they are evidently present and play a part in the biology of colonisation, probably donating DNA to as well as receiving it from the gene pool available in the nasopharyngeal niche.

Further detailed studies of colonisation in young children are clearly needed if the mechanisms of transmission, interruption of which seems to be critical for current vaccination strategies, and of development of disease, the predictors of which remain obscure, are to be understood.

## Supporting Information

S1 Fig Distribution of serotype density and log-normal fit. The empirical probability density function (pdf) of serotype density (bars), compared to the fitted log-normal distribution (red line), plotted on a semi-logarithmic scale, showing a reasonable fit.
(TIF)
