# Supplementary figures and images for: Pneumococcal Serotypes Colonise the Nasopharynx in Children at Different Densities
Source: PLoS One. 2016 Sep 29;11(9):e0163435. doi: 10.1371/journal.pone.0163435 (PMC5042462; doi:10.1371/journal.pone.0163435)

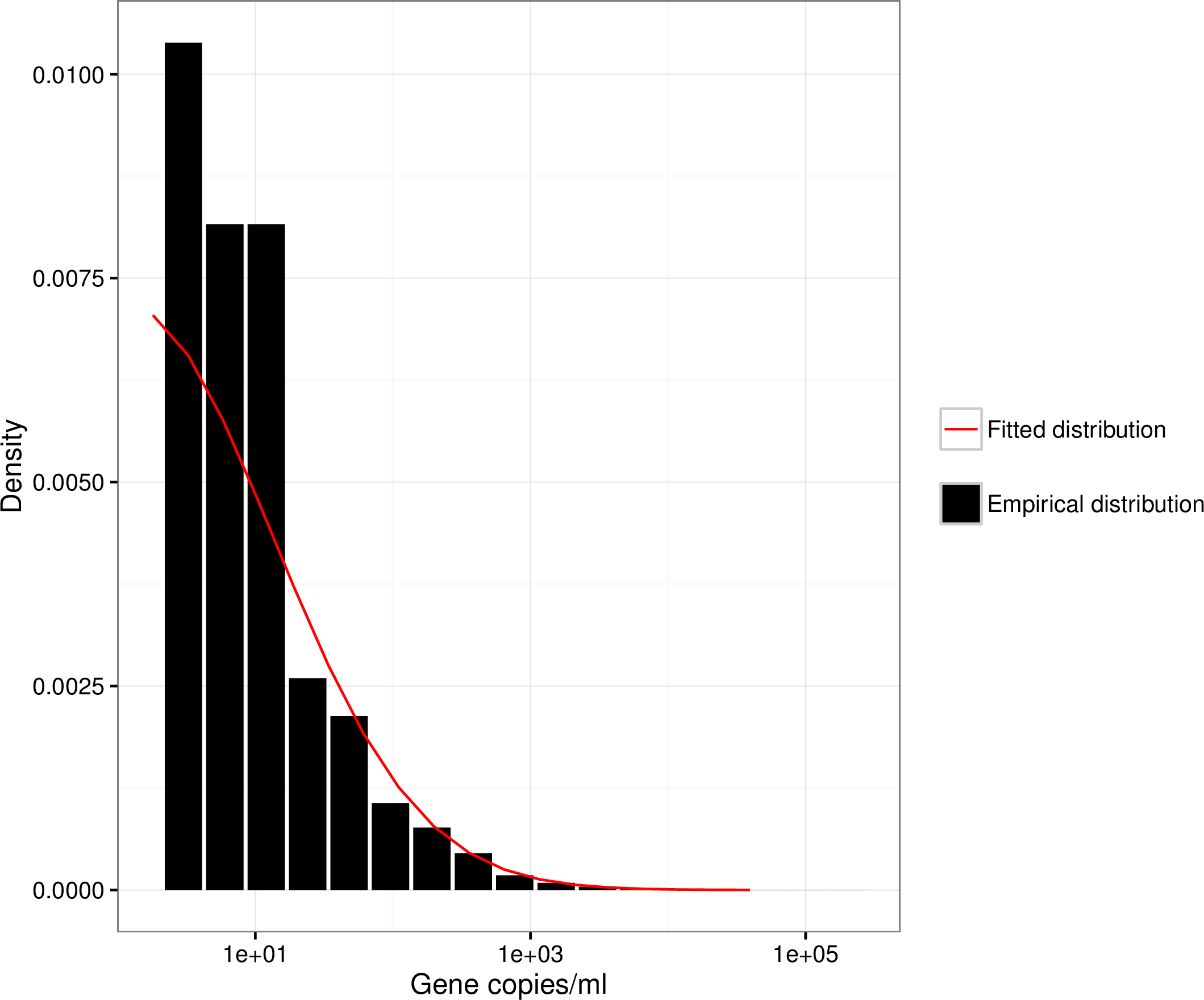

Supplement: S1 Fig — The empirical probability density function (pdf) of serotype density (bars), compared to the fitted log-normal distribution (red line), plotted on a semi-logarithmic scale, showing a reasonable fit. (TIF) [file pone.0163435.s001.tif]
